# “Liquid biopsy” - extracellular vesicles as potential novel players towards precision medicine in asthma

**DOI:** 10.3389/fimmu.2022.1025348

**Published:** 2022-11-17

**Authors:** Dominika Ambrożej, Anna Stelmaszczyk-Emmel, Małgorzata Czystowska-Kuźmicz, Wojciech Feleszko

**Affiliations:** ^1^ Department of Pediatric Pneumonology and Allergy, Medical University of Warsaw, Warsaw, Poland; ^2^ Doctoral School, Medical University of Warsaw, Warsaw, Poland; ^3^ Department of Laboratory Diagnostics and Clinical Immunology of Developmental Age, Medical University of Warsaw, Warsaw, Poland; ^4^ Chair and Department of Biochemistry, Medical University of Warsaw, Warsaw, Poland

**Keywords:** asthma, extracellular vesicle, exosome, precision medicine, intercellular communication, microRNA, allergy, inflammation

## Abstract

Extracellular vesicles (EVs) have emerged as vital mediators in intracellular communication in the lung microenvironment. Environmental exposure to various triggers (e.g., viruses, allergens) stimulates the EV-mediated cascade of pro-inflammatory responses that play a key role in the asthma pathomechanism. This complex EV-mediated crosstalk in the asthmatic lung microenvironment occurs between different cell types, including airway epithelial cells and immune cells. The cargo composition of EVs mirrors hereby the type and activation status of the parent cell. Therefore, EVs collected in a noninvasive way (e.g., in nasal lavage, serum) could inform on the disease status as a “liquid biopsy”, which is particularly important in the pediatric population. As a heterogeneous disease, asthma with its distinct endotypes and phenotypes requires more investigation to develop novel diagnostics and personalized case management. Filling these knowledge gaps may be facilitated by further EV research. Here, we summarize the contribution of EVs in the lung microenvironment as potential novel players towards precision medicine in the development of asthma. Although rapidly evolving, the EV field is still in its infancy. However, it is expected that a better understanding of the role of EVs in the asthma pathomechanism will open up new horizons for precision medicine diagnostic and therapeutic solutions.

## 1 Introduction

Extracellular vesicles (EVs) are heterogenous membranous nanoparticles secreted from every cell type in our body, varying in size, morphology, and content (1). EVs carry a multitude of intracellular bioactive molecules between cells, including nucleic acids (e.g., DNA, messenger RNA (mRNA), and non-coding RNA), lipids, and proteins (2). Consequently, EVs play a vital role in intercellular communication (2, 3). It is well-established that, for instance, the composition of EV-associated RNA mirrors the type and activation status of their parent cell (4). Therefore, collecting EVs could inform on the disease status of the cells noninvasively, without removing the cells from the tissues, serving as a “liquid biopsy” (5).

EVs can be found in all human body fluids, taking part in regulation of various systemic processes such as immune function and local modulation of organ-specific reactions (6). Over the past decade, there has been a boost in EVs exploration across the scientific community, including the role of EVs in the complex lung microenvironment (7, 8).

Currently, no single optimal EVs’ isolation method is recommended, since it depends on the desired balance between recovery and specificity and EV end use (e.g., basic vs. clinical research), however the most popular are ultracentrifugation and size-exclusion chromatography. According to MISEV2018, EV separation/isolation procedures should be reported in detail and multi-step characterization is needed to attribute reported function or a biomarker to EVs (1).

Rapidly accumulating evidence from *in vitro*, *in vivo*, and human studies has demonstrated that EVs have a potential role in asthma diagnostic options (9, 10). Asthma is a major chronic inflammatory lung disease affecting the quality of life of people of all ages worldwide, characterized by reversible airway obstruction causing dyspnea and cough (11, 12). Due to the heterogeneity of the disease (with various endotypes/phenotypes), there are still gaps to be filled in terms of improving patient education, implementing new diagnostics, and personalized case management (13, 14). In the asthma inflammatory microenvironment, both resident cells (e.g., epithelial, endothelial cells, fibroblasts) and inflammatory cells (e.g., eosinophils, mast cells, T cells) interact and exchange soluble mediators (15). Thus, the emerging EV field opens up new horizons to understanding asthma pathomechanism and offers new targets for precision medicine therapies.

Here, the recent updates on the role of EVs in the asthma pathomechanism in a cell type-specific manner will be discussed.

## 2 Types of extracellular vesicles in asthma

### 2.1 Epithelial cell-derived extracellular vesicles

**Figure 1:**
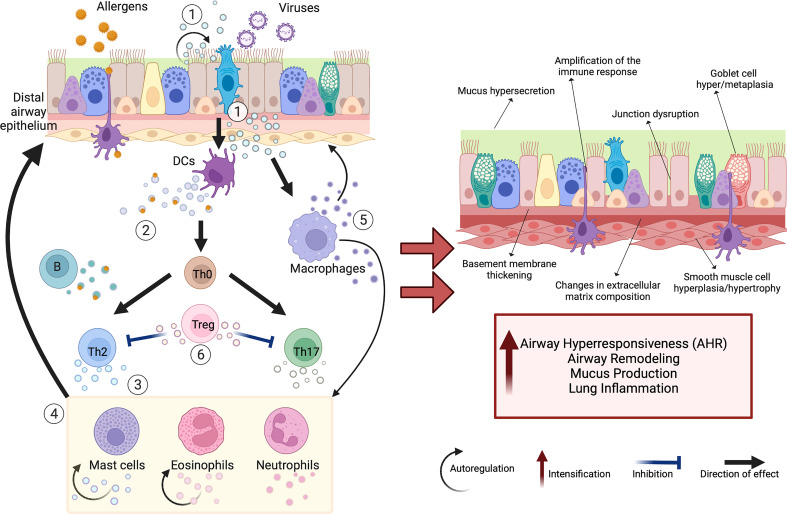
Schematic view of extracellular vesicles (EVs) as mediators of cellular interactions in the lung microenvironment. The complex EV-mediated crosstalk in the lung microenvironment occurs between many different cell types, including airway epithelial cells (AECs) and immune cells. Secreted EVs transfer specific cargo (e.g., proteins, miRNAs, mitochondria), which modulates the activity of target cells and can support tissue homeostasis or promote chronic respiratory changes. The exposure of airway epithelium to various environmental triggers (e.g., viruses, allergens) stimulates increased release of EVs with changed cargo composition that plays a critical role in asthma pathomechanism (including airway remodeling, airway hyperresponsiveness (AHR), mucus hypersecretion, increased lung inflammation). These EVs act in several ways (1): AEC-derived EVs target dendritic cells (DCs), macrophages, and themselves, promoting Th2- and Th17-polarized immune activation (2). DC-derived and B-cell-derived EVs can induce T-cell responses and serve as “antigen-presenting units” (3). T-cell-derived EVs cause Th2 skewing, eosinophil, neutrophil, and mast cell activation (4); that cells in turn produce EVs driving airway remodeling, and supply nitric oxide and reactive oxygen species, increasing the migration of other eosinophils and mast cells to the inflammation site (5). Macrophage-derived EVs also participate in airway remodeling, synthesizing leukotrienes and recruiting granulocytes to the inflammation site (6). In contrast, regulatory T cell (Treg)-derived EVs initiate anti-inflammatory activities. Created with BioRender.com.

Airway epithelial cells (AECs) play an important role in asthma development, both as a barrier and as modulators of the immune response, including innate mucosal defense (16, 17). AECs released EVs apically and basally, with side-specific functions of the miRNA cargo (**Figure 1**) (18, 19). Epithelial remodeling processes such as mucin hypersecretion can be significantly altered by the changed miRNA profile of AEC-EVs (16). In a recent *in vitro* study, stimulation with T2 cytokines of human AECs upregulated the release of EV proteins involved in chronic airway inflammation and decreased the expression of the antimicrobial peptide S100A7, suggesting that EVs mediate endotype-specific mechanisms related to asthma (20–22). Under T17 immune response conditions, EV-associated proteins increased neutrophil recruitment and promote neutrophilic airway inflammation (20).

Despite the current knowledge on various AECs phenotypes, we are still unable to easily detect or monitor airway epithelial cell viability/dysfunction in biosamples possessed in minimally invasive procedures, e.g., in nasal lavages, or exhaled breath condensate (EBC) (23, 24).

Some newly detected EV-related proteins were linked to asthma pathology: ezrin, contantin-1 (CNTN1) and Plexin B2 (PLXNB2). Firstly, ezrin as part of cytoskeletal elements of AEC, derives directly to EVs from AECs. EV-associated ezrin released by AECs contributed to IL-13-induced epithelial damage *via* the TNF-α-dependent pathway and was proposed as a biomarker of asthma control (25, 26). Secondly, CNTN1 promoted a Th2- and Th17-polarized immune activation through the Notch-2 pathway along with smooth muscle hyperresponsiveness (AHR) and mucus production in cell and mouse model (27). Thirdly, PLXNB2, a natural CD100 ligand, was released in AECs-EVs and augmented neutrophilic and monocytic airway inflammation in mice by activating macrophages *via* cleavage of CD100 by MMP14 (28). Furthermore, a lower expression of miR-34a, miR-92b, and miR-210 was found in EVs in nasal lavages from asthmatic children, and was associated with an obstruction of large (FEV_1_FVC_%pred_) and small airways (FEF_25-75%pred_) (18).

### 2.2 Immune cells-derived extracellular vesicles

#### 2.2.1 EVs from mast cells

Mast cells (MCs) have been recognized as active participants in innate as well as specific immune responses (29). Their EVs were shown to play a role in positive immune regulation, including recruiting B and T cells to the lungs and facilitating the priming of naïve T cells in an *in vitro* and a mouse model (30–33). EVs from bone marrow-derived MCs, carrying high-affinity IgE receptors (FcϵRI), can bind to free IgE *via* FcϵRI, induce an anti-IgE effect, thus decreasing IgE levels and inhibiting the allergic cascade (34). In a mouse model of allergic asthma, these EVs modulated not only airway inflammation and AHR, but also partially the remodeling in chronic asthma (34). On the other hand, CC chemokine receptor (CCR1)-rich MCs-EVs could transfer CCR1 to other MCs *in vivo*, enhancing the co-activation of high-affinity IgE receptors (FcϵRI) with CCR1 (35, 36).

Furthermore, it was proven that MCs-EVs take part in the modulation of oxidative stress. In asthmatic mice, miR-21 released in MCs-EVs promotes oxidative stress and inflammatory responses *via* the DDAH1/Wnt/β-catenin signaling axis (37). Moreover, mouse MCs-EVs exposed to oxidative stress had a different mRNA profile, transferring resistance to further oxidative damage to recipient cells; however, this mechanism is far from being elucidated (38).

#### 2.2.2 EVs from dendritic cells

The lung-resident dendritic cells (DCs) participate in asthma development, as they have a pivotal role in establishing an allergen-specific Th2 response in the airways after stimulation with epithelial alarmins (39, 40). It has been shown that activated DCs released EVs containing various protein ligand-like OX40L that induced the proliferation of CD4+ T cells, elevated the level of IL-4, and drove Th2 differentiation *in vitro* (41). Furthermore, DC-derived EVs provided enzymes for the biosynthesis of leukotrienes (LTs), key pro-inflammatory mediators important in the pathogenesis of asthma, to smooth muscle cells. Besides, these EVs contained chemotactic eicosanoids and promoted granulocyte migration *in vitro* (42).

Majority of the studies on DCs-EVs were conducted using either bone marrow-derived or monocyte-derived DCs. Recently, it has been recognized that pulmonary DCs present different DCs subsets, including conventional types 1 and 2 DCs and plasmacytoid DCs, each playing a varying role in asthma pathogenesis (43). This evidence emphasizes the need for researchers to further specify the origin and effect of DCs-EVs in a subtype manner.

#### 2.2.3 EVs from macrophages

The growing appreciation of macrophage plasticity and polarization in asthma pathogenesis reflects the pro-inflammatory properties of M1 polarized macrophages and the anti-inflammatory properties of M2 polarized macrophages (44–46). M2-like alveolar macrophages were reported to secrete suppressor of cytokine signaling (SOCS)-1 and SOCS-3 proteins within EVs (47, 48). Epithelial cells exposed to these EVs presented alleviated cytokine signaling *via* the JAK-STAT pathway activation. Thus, impaired delivery of SOCS proteins through EVs could serve as a significant mechanism in the dysregulated cytokine responses in asthma.

Under the stress condition, rat alveolar macrophages produced EVs carrying high levels of several microRNAs, including miR-21-5p involved in the oxidative stress. The EVs transported miR-21-5p to tracheal epithelial cells and promoted airway remodeling through the TGF-β1/Smad signaling pathway by targeting Smad7 (49). Similarly to DCs, EVs released from macrophages took part in the biosynthesis of LTs and granulocyte migration *in vitro* (42).

#### 2.2.4 EVs from eosinophils

For decades, eosinophilia has been recognized as one of the prominent features of allergic asthma, and eosinophils are linked with the so-called T2-high asthma endotype (50). Eosinophils from asthmatic patients produced higher EV levels than those from healthy subjects, and these EVs contained molecules relevant to human asthma, such as EPO (eosinophil peroxidase), MBP (major basic protein), and eosinophil cationic protein (EPC) (51). Furthermore, these EVs induced epithelial cell apoptosis and smooth muscle cell proliferation, which are fundamental aspects of asthma pathogenesis (52). Moreover, they demonstrated properties to autoregulated and promoted eosinophil function in asthmatic inflammation by producing nitric oxide and reactive oxygen species (53). Moreover, eosinophil-derived EVs acted *in vivo* as a chemotactic factor for eosinophils due to the expression of adhesion molecules, such as intercellular adhesion molecule (ICAM)-1 and integrin α2 (53). Altogether, eosinophilic EVs, acting in a feedback loop for their short-term lived parent cells, may prolong the inflammatory cellular infiltration and muscle and epithelial remodeling.

#### 2.2.5 EVs from neutrophils

Neutrophilic airway infiltration is observed in non-allergic asthma, often in severe cases with poor response to corticosteroid treatment (54, 55). Proteomic composition of neutrophil-derived EVs, released spontaneously and upon lipopolysaccharides (LPS)-stimulation, significantly varied (56). The EVs from LPS-stimulated equine neutrophils contained higher levels of thrombospondin-1 and S100A9, and lower levels of neutrophil gelatinase–associated lipocalin and serpin peptidase inhibitor. This analysis provided evidence of neutrophils-derived EVs’ contribution to tissue inflammation, apoptosis modulation, and proliferation of smooth muscle cells (56). Therefore, it supported the involvement of these EVs in the progression of asthma and the promotion of airway remodeling in severe and corticosteroid-insensitive patients with asthma. In another study, EV transfer of activated neutrophil-derived long non-coding RNA CRNDE was suggested to promote proliferation and migration of airway smooth muscle cells in asthma (57). Thereby, *in vivo* silencing of CRNDE reduced the thickness of bronchial smooth muscle in asthmatic mice.

#### 2.2.6 EVs from T- and B-cells

T cells are of great importance in the adaptive immune responses during the asthma pathomechanism by participating in IgE antibody class switching, Th2 skewing, eosinophil and mast cell activation. A recent study, using a proteomic approach, demonstrated that EVs from activated human T cells had enhanced expression of the RAS/MAPK signaling pathway proteins, which induced ERK kinase phosphorylation in recipient immune cells *in vitro* (58). By contrast, regulatory T cells (Tregs) are a subpopulation of T cells that aim to maintain immunological tolerance, prevent autoimmunity, and limit other immune responses (59). They achieve this through various mechanisms, including Treg-derived EVs. Upon LPS stimulation, Tregs-derived EVs were found to transfer miR-150-5p and miR-142-3p to DCs, modulating DCs cytokine secretion towards an anti-inflammatory profile of increased IL-10 and decreased IL-6 (59). In another *in vitro* study, following activation of Tregs, CD73-expressing Treg-derived EVs demonstrated their suppressive activity through the production of adenosine (60).

Th2-mediated inflammation is also promoted by B cell EVs that carry allergen peptides on MHC molecules. This antigen-presenting property of B cell-derived EVs was shown *in vitro.* Birch-allergen (Bet v 1)-loaded-B-cell EVs induced T cell proliferation and secretion of IL-5 and IL-13 cytokines, key signals in driving airway inflammation and remodeling in asthma (61).

### 2.3 Mesenchymal-stem-cell-derived extracellular vesicles

Mesenchymal stem cells (MSCs) refer to a group of cells from bone marrow, adipose tissue and umbilical cord, that has the capacity for adherent growth (62, 63). MSCs are widely used in cell-based therapy due to their remarkable ability for proliferation, differentiation, and immune regulation. However, translating MSCs into clinic remains more difficult than expected (64). Currently, there is accumulating evidence on a promising role of MSCs-derived EVs - rather than MSCs itself, in asthma therapy. The summary of available data associating various MSCs-derived EVs with possible therapeutic options for asthma is presented in **Table 1** and is discussed in more detail in chapter 3.3.

**Table 1:** Mesenchymal-Stem-Cell-Derived extracellular vesicles (MSC-EVs) and their reported role in attenuating asthma pathomechanisms.

Cell type releasing EVs	EV isolation and characterization method	EV molecular signatures assessed	Key findings	Reference
Mouse ASCs	Filtration and differential ultracentrifugation; TEM, WB, NTA	CD81, CD40, calnexin	Intranasal administration of ASC-derived EVs to asthmatic mice reduced allergic airway inflammation (including the total inflammatory cells and eosinophils in the BALF), AHR, and also improved lung pathology.	Mun et al., 2021 (65)
Mouse ASCs	Filtration and differential ultracentrifugation; TEM, WB	Positive for: CD29, CD90, CD44, CD105, Negative for: CD34, vWF	Intravenous administration of mmu_circ_0001359-enriched ASC-derived EVs attenuated airway remodeling in asthmatic mouse model by targeting FoxO1 mediated M2-like macrophage polarization.	Shang et al., 2020 (66)
hBM-MSCs	Differential ultracentrifugation; TEM, WB, NTA	CD81, TSG101	Intravenous administration of hBM-MSC-derived EVs suppressed proliferation of bronchial smooth muscle cells and lung injury in asthmatic mice through the miR-188/JARID2/Wnt/β-catenin axis.	Shan et al., 2022 (67)
hBM-MSCs	Differential ultracentrifugation; TEM, WB	CD9, CD81	hBM-MSC-derived EVs promoted Tregs proliferation and immunosuppression capacity by upregulating suppressive cytokines IL-10 and TGF-β1 in PBMCs of asthmatic patient.	Du et al., 2018 (68)
hBM-MSCs	Differential ultracentrifugation; TEM, WB	CD9, CD63, CD81	hBM-MSC-derived EVs miR-1470 promoted the differentiation of CD4+CD25+FOXP3+ Tregs isolated from peripheral blood of asthmatic patients by inducing the expression of P27KIP1.	Zhuansun et al., 2019 (69)
hBM-MSCs	Differential ultracentrifugation; TEM, NTA, flow cytometry	CD63, CD9, CD81	*In vitro* experiments showed that hBM-MSC-derived EVs modify DC function, and that delivery of miR-21-5p *via* EVs may be an important mechanistic pathway in asthma pathogenesis.	Reis et al., 2018 (70)
hUC-MSCs	Differential ultracentrifugation; WB, NTA	TSG101, HSP70, collagen-1, α-SMA, TGF-β1, HIF-1α, Gapdh, β-actin	Intravenous administration of hypoxic hUCMSC-derived EVs attenuated allergic airway inflammation and airway remodeling in chronic asthma mice more effectively than normoxic hUCMSC-derived EVs.	Dong et al., 2021 (71)
hUC-MSCs	Membrane affinity columns; TEM, NTA, flow cytometry	CD63, CD81	Intratracheal administration of hUCMSC-derived EVs ameliorated severe, steroid-resistant asthma in a mouse model by moderating inflammation, which is achieved by reshaping macrophage polarization *via* inhibition of TRAF1.	Dong et al., 2021 (72)
human iPSC-MSCs	Anion-exchange chromatography; TEM, flow cytometry	CD63, CD9, CD81, CD44, CD146, CD73, CD90, CD105	Intravenous administration of human iPSC-MSC-derived EVs reduced ILC2-dominant allergic airway inflammation at least partially through miR-146a-5p in asthmatic mouse model.	Fang et al., 2020 (73)
human iPSC-MSCs	Anion-exchange chromatography; TEM, flow cytometry	CD63, CD9, CD81, ALIX, TSG101, Calnexin	Intravenous administration of human iPSC-MSC-derived EVs ameliorated Th2-dominant allergic airway inflammation through immunoregulation on pulmonary macrophages in asthmatic mouse model.	Fang et al. (74)

ACSs, adipose stem cells; AHR, airway hyperresponsiveness; BALF, bronchoalveolar lavage fluid; DC, dendritic cell; EVs , extracellular vesicles; hBM-MSCs, human bone marrowmesenchymal stem cells; hUC-MSCs ,human umbilical cord mesenchymal stem cells; iPSC-MSCs, Mesenchymal Stem Cells derived from Induced Pluripotent Stem Cells; IL, interleukin; ILC2, group 2 innate lymphoid cells; NTA, nanoparticle tracking analysis; PBMCs, peripheral blood mononuclear cells; TEM, transmission electron microscopy; TRAF1, tumor necrosis factor receptorassociated factor 1; Tregs, regulatory T cells; TSG101, tumor susceptibility 101; vWF, vonWillebrand Factor; WB, Western Blot.

The above discussed role of EVs in asthma pathobiology, derived from major cell types within the lung microenvironment, is summarized in **Figure 1**.

## 3 Discussion and future research directions

Since the discovery of EVs decades ago, tremendous progress has been made in the deciphering how they are involved in intracellular communication, impacting various physiological and pathological events. However, this novel field is still in its infancy. To date, in asthma research, the EVs have been successfully isolated and characterized from several human biofluids, obtained *via* non-invasive methods, including saliva (75), nasal lavage fluid (18), EBC and sputum (76). Despite our growing knowledge on asthma heterogeneity, there is an unmet need for development of molecular markers guiding further the precision medicine approach, both in asthma diagnostic and therapeutic management. Notably, specially pediatricians worldwide face a great number of obstacles in the management of their asthmatic patients (77). It includes a precarious invasive collection of biological samples, lack of precise diagnostic tests, as children are often unable to perform lung function tests, and the common failure to recognize the variability of the course of asthma. This frequently leads to young asthma patients being underdiagnosed, undertreated, and inadequately controlled (78).

### 3.1 Role of EVs in asthma pathology

Viral respiratory infections, particularly with the rhinovirus (RV) and the respiratory syncytial virus (RSV), are major causes of asthma development and exacerbations (79, 80). EVs offer new insight into the viral-induced chronic sequelae in the lung microenvironment. RSV-infected APCs were found to initiate EVs production that contain agents promoting inflammatory cytokine production *in vitro* in an alveolar epithelial cell line culture through IP-10, CCL2, and CXCL10 release (81). Interestingly, RSV infection of AECs was associated with significant changes in EVs RNA content (e.g., upregulated EVs-miRNAs: hsa-mir-6087, hsa-let-7e, hsa-miR-182-5p, hsa-miR-181b-5p; downregulated EVs-miRNAs: hsa-mir-223, hsa-mir-2964a, hsa-mir-205, hsa-mir-143) (81). Furthermore, EVs derived from virus- infected cells contain RSV components but do not transmit RSV infection (81). Moreover, these EVs induce pro-inflammatory mediator secretion in uninfected bystander cells, thereby impacting the additional way to modulate the immune responses during infection (81). Another comparative analysis of the airway secretory microRNAome in children under the age of three indicated that RV infection is associated with airway secretion of EVs enclosing miR-155, which *in silico* was predicted to regulate antiviral host immunity (82). Additionally, two distinct components of the inflammatory pathway regulating the immune response were revealed following RV infection and TLR3 (not TLR7) stimulation in asthmatic AECs. The first of these components highlighted a Tenascin-C protein release, including its upregulated expression in nasal lavage fluid (83). The second noted a secretion of EVs with a pro-inflammatory effect on AECs. By contrast, umbilical cord MSCs-derived EVs were shown to possess antiviral activities against other common human respiratory viruses (84).

Recent data revealed also the role of airway and even gut microbiota in asthma pathogenesis, and microbiota-derived EVs are emerging as the linking factor between microbiota and allergic reactions and asthma and are discussed in more detail elsewhere (85, 86).

### 3.2 EV-based asthma biomarkers

Recent evolution of high-throughput sequencing technology with advanced analytical methods enables precisely tracking changes in EVs’ cargo composition and delineating complex interactions within molecular networks of asthma endotypes. For instance, circulating and/or EVs- related non-coding RNAs have been introduced as the novel, valuable biomarkers for different pathological conditions, including asthma. In particular, a promising makrer – the long non-coding RNA (lncRNA) impacts a wide range of biological processes (e.g., transcriptional activation, transcriptional interference by competitively inhibiting the effect of miRNA on downstream mRNA) (87). At this moment, the very first steps were made to explore the lncRNA-miRNA-mRNA regulatory network in T2-high asthma (57, 88). For example, PCAT19 was suggested as a lncRNA that may serve as a promising immune-related biomarker to distinguish between T2-high and T2-low asthma; however, it was not studied as a part of the EVs content (88). A novel study associated the levels of four serum EV-miRNAs (miR-21-5p, miR-126-3p, miR146a-5p, miR-215-5p) with the severity of asthmatic in adults (89). miR-21-5p and miR-126-3p, involved in Th1/Th2 differentiation, were specifically augmented in T2-high asthma. By contrast, IL-6-high patients with MSA, which were older, more obese, with higher neutrophil and basophil counts and TNF levels, manifested a decrease of miR-21-5p, miR-126-3p and miR-146a-5p. Interestingly, the researchers observed a trend towards a decreased expression of all studied miRNAs in mild asthmatics compared to healthy controls, probably due to the effect of inhaled corticosteroids (89). More research is clearly needed to clarify the role of EV-miRNAs in asthma endotyping/phenotyping and for undergoing treatment, and the current data are too limited to speculate about their possible use in clinical practice.

### 3.3 EV-based therapeutical strategies for asthma

Compared with other commonly used drug delivery carriers, such as liposomes, EVs have the advantages of high internal targeting ability, low immunogenicity, high modification flexibility and high biological barrier permeability, which open up an exciting avenue for modern drug delivery (63, 90). For example, experimentally engineered nanoparticles – extracellular vesicle’s membrane from M2 macrophages combined with a lncRNA named methyltransferase 3A opposite strand (Dnmt3aos) smart silencer wrapped in a polylactic acid-glycolic acid (PLGA) copolymer – have been demonstrated to target M2 macrophages *in vitro* and *in vivo* and reduce airway inflammation, while not suppressing the overall immune function of the host (91). Therefore, these innovative nanoparticles can be an attractive candidate for the potential immunotherapy for asthma.

MSCs-EVs are the hot spot of current research as they have been identified to inherit the anti-inflammatory and immunomodulatory properties of stem cells, inducing the M2 polarization of macrophages, reducing inflammation, while avoiding the disadvantage of stem cells such as tumorigenicity (46). Importantly, MSCs-EVs present a specificity of the EV/macrophage axis (46). In allergic rhinitis, human MSCs-EVs could inhibit the differentiation of Th2 cells *via* the regulation of the miR-146a-5p/SERPINB2 pathway (92), which shall be extrapolated for allergic inflammation of lower airways.

Several potential therapeutic strategies for asthma were sparked by an expansion in multi-omic and EV research. Lee et al. suggested immunoregulatory effects of *Lactococcus lactis*-derived EVs by shifting the immune responses from Th2 to Th1, mediated by DCs activation in allergic asthma (93). Studies on EV-based therapeutics for asthma treatment are highly anticipated. Preliminary findings reported an opportunity for inhalable dry powder mRNA vaccines based on EVs, which may pave the way to decreasing the risk for severe early-life respiratory infections like bronchiolitis and, consequently, de-risk further asthma development (79, 94, 95). Also, microbial EVs have recently been considered promising diagnostic and therapeutic tools for various inflammatory diseases (86).

### 3.4 Challenges and perspectives

Despite the magnitude of reports on EVs’ advantages as diagnostic markers or therapeutic agents, there is still much to learn about their features, biological functions, and potential particle-particle interaction (96). Among the studies gathered in this review, considerable diversity in methodology could be noticed. Another key thing to remember is that most of the findings were reported by individual *in vitro* and animal studies, whereas studies on clinical samples from asthma patients are less numerous. With growing heterogeneity in EVs collection, isolation and characterization, clinical and basic researchers recognize the need for a more standardized scalable sample collection and processing methodology to obtain reproducible results. Work is underway to deliver an update in 2022 on detailed Minimal information for studies of extracellular vesicles 2018 (MISEV2018) recommendations by the International Society for Extracellular Vesicles (ISEV) (1). Moreover, from a technical point of view, long-term storage and a freeze–thaw cycle of biosamples may lead to disturbance of various structures. Evidence suggests storage of EVs isolated from bronchoalveolar lavage fluid (BALF), could destabilize the surface characteristics, morphological features, and protein content of these EVs (97). Due to the protein leakage from the EVs into the supernatant, around 50% of protein composition showed differences in abundance in BALF-EVs as a result of the storage at both +4°C and −80°C. Thus, it was proposed that airway EVs should be best analyzed immediately after isolation. This constitutes another critical challenge for the implementation of EVs into daily clinical practice.

## 4 Conclusions

Taken together, EVs represent a potential novel player towards precision medicine in the diagnosis and treatment of asthma. Although rapidly evolving, the EV field is still in its initial stage. In the near future, it is expected that research efforts in this area will enable further understanding of the role of EVs in the complex mechanisms underlying asthma pathogenesis; hence, providing a solid background for precision medicine diagnostic and therapeutic solutions.

## Author contributions

DA prepared the first draft of the manuscript, designed the figure and the table, and made the editing following other authors’ comments. AS-E, MC-K and WF reviewed the first draft of the manuscript, gave directions around the table and the figure design and worked on the editing of manuscript to help reach the final version. All authors contributed to the article and approved the submitted version.

## Funding

This research was funded by the National Science Centre, Poland under grant PRELUDIUM 20 2021/41/N/NZ5/02950 (DA). The funds for open access publication fees were received from Medical University of Warsaw.

## Conflict of interest

The authors declare that the research was conducted in the absence of any commercial or financial relationships that could be construed as a potential conflict of interest.

## Publisher’s note

All claims expressed in this article are solely those of the authors and do not necessarily represent those of their affiliated organizations, or those of the publisher, the editors and the reviewers. Any product that may be evaluated in this article, or claim that may be made by its manufacturer, is not guaranteed or endorsed by the publisher.
